# PIAS family in cancer: from basic mechanisms to clinical applications

**DOI:** 10.3389/fonc.2024.1376633

**Published:** 2024-03-25

**Authors:** Xiaomeng Li, Azhar Rasul, Farzana Sharif, Mudassir Hassan

**Affiliations:** ^1^ KingMed School of Laboratory Medicine, Guangzhou Medical University, Guangzhou, Guangdong, China; ^2^ Department of Zoology, Government College University Faisalabad, Faisalabad, Pakistan

**Keywords:** PIAS, SUMOylation, transcriptional factor, signal transducers, activators of transcription factor (STAT)

## Abstract

Protein inhibitors of activated STATs (PIAS) are proteins for cytokine signaling that activate activator-mediated gene transcription. These proteins, as versatile cellular regulators, have been described as regulators of approximately 60 proteins. Dysregulation of PIAS is associated with inappropriate gene expression that promotes oncogenic signaling in multiple cancers. Multiple lines of evidence have revealed that PIAS family members show modulated expressions in cancer cells. Most frequently reported PIAS family members in cancer development are PIAS1 and PIAS3. SUMOylation as post-translational modifier regulates several cellular machineries. PIAS proteins as SUMO E3 ligase factor promotes SUMOylation of transcription factors tangled cancer cells for survival, proliferation, and differentiation. Attenuated PIAS-mediated SUMOylation mechanism is involved in tumorigenesis. This review article provides the PIAS/SUMO role in the modulation of transcriptional factor control, provides brief update on their antagonistic function in different cancer types with particular focus on PIAS proteins as a bonafide therapeutic target to inhibit STAT pathway in cancers, and summarizes natural activators that may have the ability to cure cancer.

## 1 The PIAS proteins

The term PIAS (protein inhibitors of activated STAT) originates due to its cellular function, as all members of this family negatively regulates the STAT (1, 2). The PIAS were primarily identified via yeast two hybrid (Y2H) technique. The PIAS proteins that are found in eukaryotes are evolutionary conserved from yeast and process versatile cellular regulatory function of approximately 60 proteins (3, 4). The PIAS family comprises four genes, generating a total of seven proteins: PIAS1, PIASxα, PIASxβ, PIAS3, PIAS3L, PIASy, and PIASyE6 (as called PIAS4) are reported in all eukaryotic and mammalian cells 19526197. In eukaryotes, PIAS proteins play a key role in activating a wide range of cellular pathways, including nuclear transport through intracellular channels, Sma- and Mad-related proteins, along with DNA damage repair through the recruitment of transcription factors (5).

## 2 Historical perspective

PIAS3 was first identified in 1997 as an inhibitor of interferon alpha (IFN-α)-induced STAT3-mediated transcriptional regulation in cytoplasmic and nuclear extracts of human and mouse cell lines (6). In 1998, additionally four members of PIAS family were isolated from JY112 B cells. Co-immunoprecipitation results indicate that PIAS1 has the ability to interact with only STAT1 to mask DNA binding domain but not with STAT2 or STAT3 (3). Not long after, the PIAS family was found to negatively control the JAK/STAT pathway to inhibit the transduction cascade (7). PIAS1 expression as a co-modulator in monkey and mouse was reported first in this study. Importantly, the co-expression of PIAS1 with androgen receptors (ARs) was found to be crucial for AR initiation and maintenance of spermatogenesis (8). PIAS3 also interacts with AR as co-regulator in prostate cancer (9). In 2001, a distinct effect of PIASy was reported as blocker of AR in prostate cancer (10). The most important discovery related to PIAS protein in 2001 is its role to catalyze the SUMOylation of various of LEF1, p53, and STAT (11). The “PINIT” domain is a well conserved domain in PIAS family consisting of 181 amino acids essential for nuclear localization of PIAS3L identified in mouse (12). PIASy, by recruiting histone deacetylase 1 (HDAC1), interacts with Smad protein and functions as a downregulator of Smad-mediated transcriptional responses (13). Further investigation determined the PIAS3 function to stimulate the Smad transcriptional activity (13). Progress in the study of PIAS focused on PIAS as a new target for anticancer therapy. Overexpression of PIAS3 reduces the STAT3 transcription in glioblastoma and ovarian cancer. Curcumin was first reported to control the activated STAT3 by affecting the expressions of PIAS and JAK/STAT suppressor genes in cancer (14).

**Figure 1:**
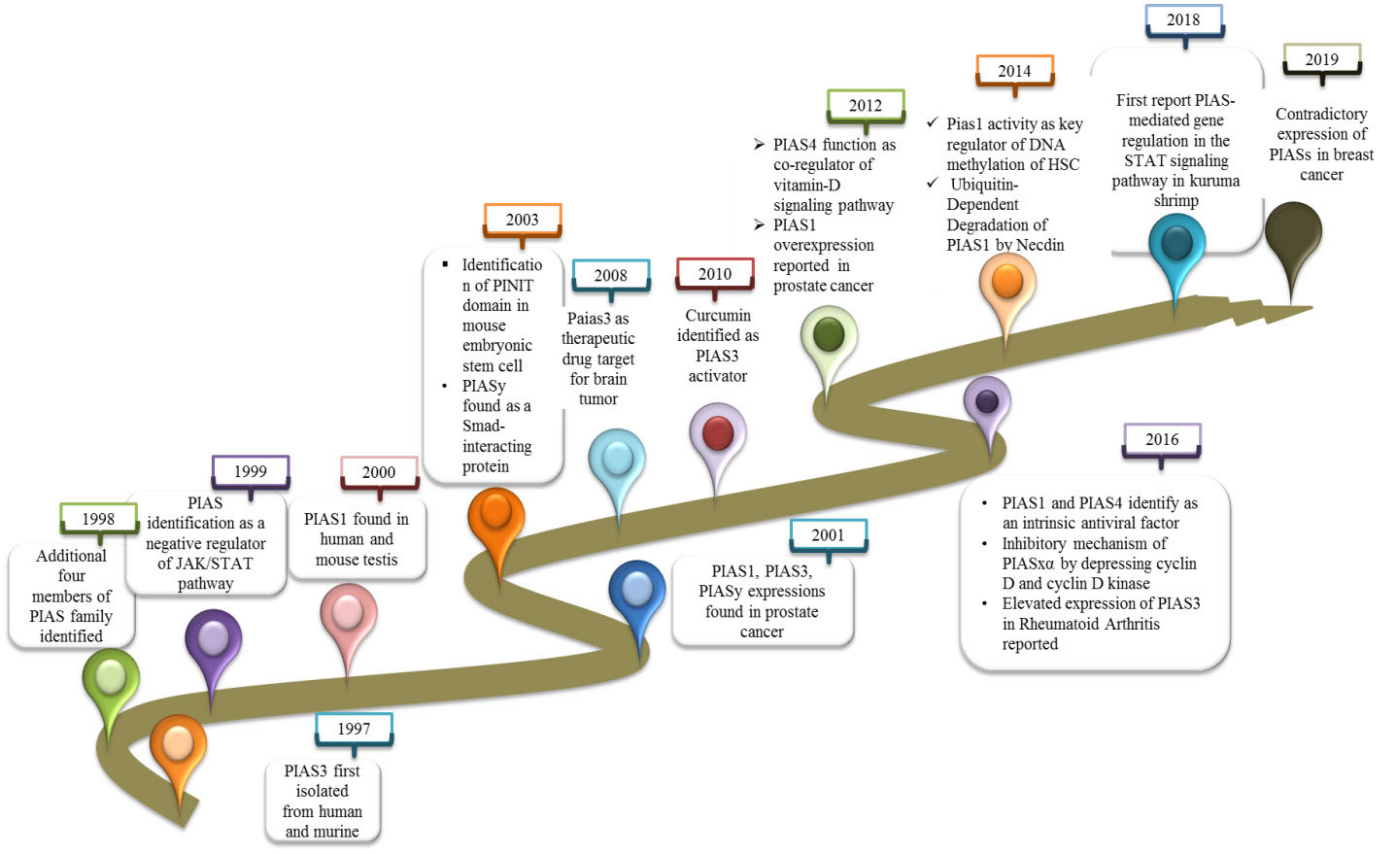
A record of discoveries related to PIAS proteins.

A further study investigated the E3-SUMO ligase activity of PIAS4 as co-regulators by inhibiting and modifying the expression of vitamin D receptor (VDR). The PIAS family was also reported as a novel interacting partner of cleavable isoforms of receptor tyrosine kinase ErbB4 ICD (15). At that time, PIAS1 overexpression was reported for first time in the nucleus of prostate cancer cells (16). The new findings recognized PIAS1 as a critical regulator of non-mutational myelo-erythroid genes inactivation in hematopoietic stem cell (HSC) and in lymphoid progenitors (17). Later on, a protein necdin was reported that overwhelmed the proapoptotic activities of PIAS1 (18). The efficacy of PIAS4 and PIAS1 function as intrinsic antiviral factor towards intracellular viral infection was investigated (19). In herpes simplex virus 1 infection, PIASy was overexpressed, which localized to nuclear domain containing viral genome. SIM domain of PIAS4 is responsible for nuclear localization in viral genome, whereas its expression that increases in replication compartments depended on SAP domain or LxxLL motif (20). Compared to other members of the PIAS family, PIAS3 is elevated in fibroblast-like synoviocytes (FLSs) and STs derived from chronic inflammatory joint rheumatoid arthritis (RA) patients (21). This discovery demonstrated the PIAS3 and another member of PIAS family, PIAS4, as positive regulators of hypoxia inducible factor (HIF-1α)-mediated transcription (22). The research supported that PIASxα was expressively lower in osteosarcoma, but the main focus was on the inhibitory mechanism of PIASxα in osteosarcoma by downregulating the important modulators of cell cycle such as cyclin D kinase in nude mouse tumor model (23). A new molecular mechanism of PIAS was reported for the very first time in kuruma shrimp (*Marsupenaeus japonicus*) as a negative regulator of transcription factor, embracing inhibition of STAT phosphorylation and translocation into the nucleus (2). PIAS family members were differentially expressed in breast cancer, as PIAe S2 and PIAS3 were downregulated, whereas PIAS4 has a contradictory trend (24) (**Figure 1**).

## 3 Structure of PIAS family

**Figure 2:**
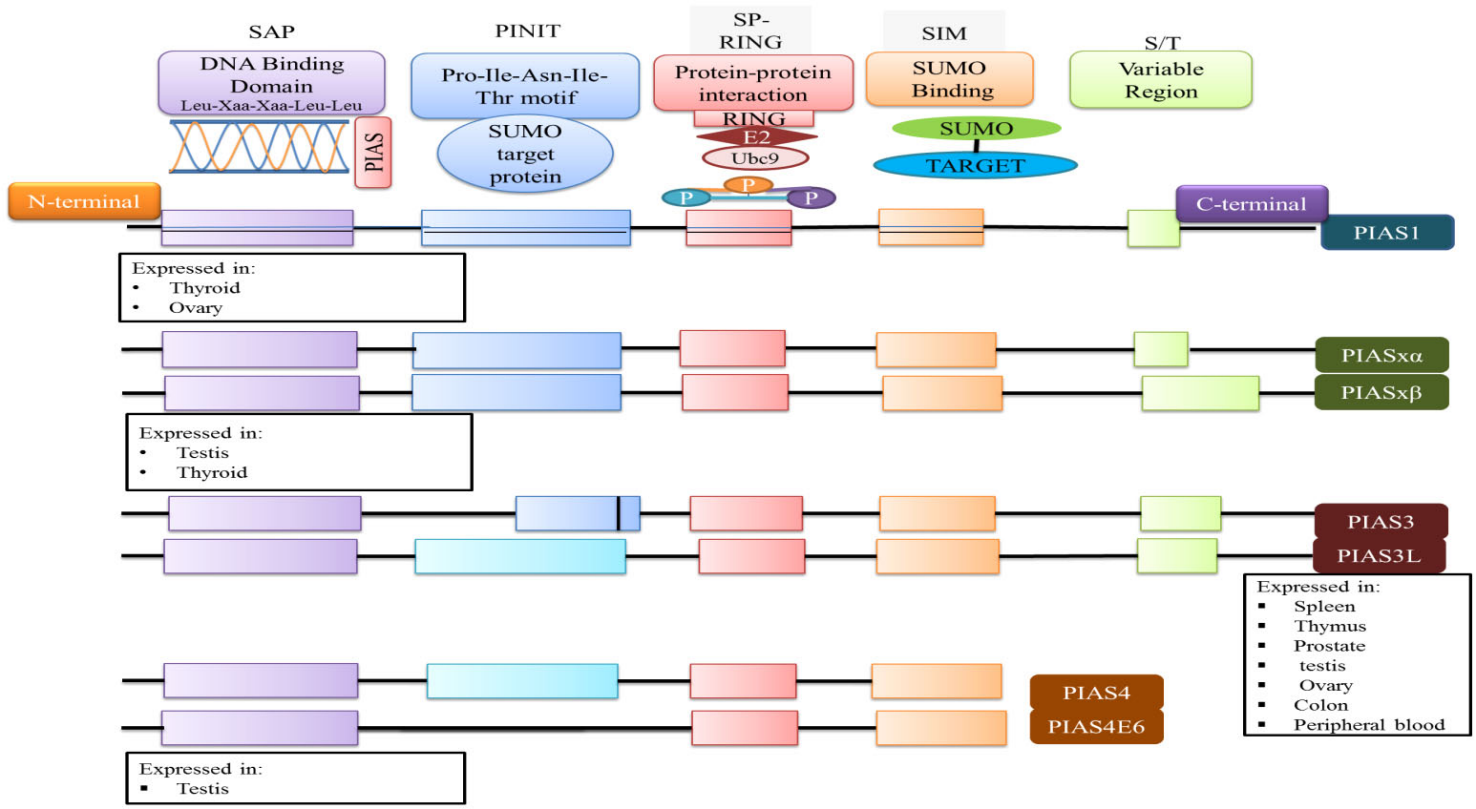
The domain structure of PIAS protein and their significance are illustrated. Each member of PIAS displays a distinct pattern of tissue expression.

The PIAS family comprises four members, which are highly homologous and shared analogous domains, but amino acid numbers vary among members of the PIAS family. PIAS1 with 651 amino acids retained the least number of residues. Genetic alteration of exon in PIAS 2, 3, and 4 give rise to their splice variants such as PIAS2ab, PIAS3L, and PIAS4E6, respectively (**Table 1**). Generally, five diverse domains have been reported in this family. Within N-terminal, PIAS protein is characterized by scaffold attachment factor (SAF)-A/B. SAP domain contains 35 amino acids along with LXXLL signature, which plays a fundamental role in A/T rich structure that facilitates protein–DNA interaction (25). SAP domain is crucial for PIASy to target lymphoid enhancer factor 1 (LEF1), suggesting its interaction with some additional SUMOylated substrate (11). The PINIT motif domain was found in PIASs, which is essential for protein localization (1). PIAS4 and its splice variant PIAS4E6 entirely lack PINIT motif. A zinc-binding domain, a central domain rich with cysteine residue, is also present. PIAS protein domains vary regarding to amino acid numbers such as PIAS3L isoform comprised of additional 35 amino acids between SAP and PINIT domain as compared to PIAS3. PIAS2a and PIAS2b diverge with respect to the length of S/T region (**Figure 2**) (1).

**Table 1:** Difference between the members of PIAS family.

Sr. No	Enzyme	Isoforms	Amino acids	References
**1**	PIAS1		651	(1, 6)
**2**	PIAS2	PIASα	572	(1, 6)
		PIASβ	621	
**3**	PIAS3		593	(1, 6)
		PIASL	628	
**4**	PIAS4		510	(1, 6)
		PIAS4E6	467	(1, 6)

## 4 PIAS protein as transcriptional regulator

**Figure 3:**
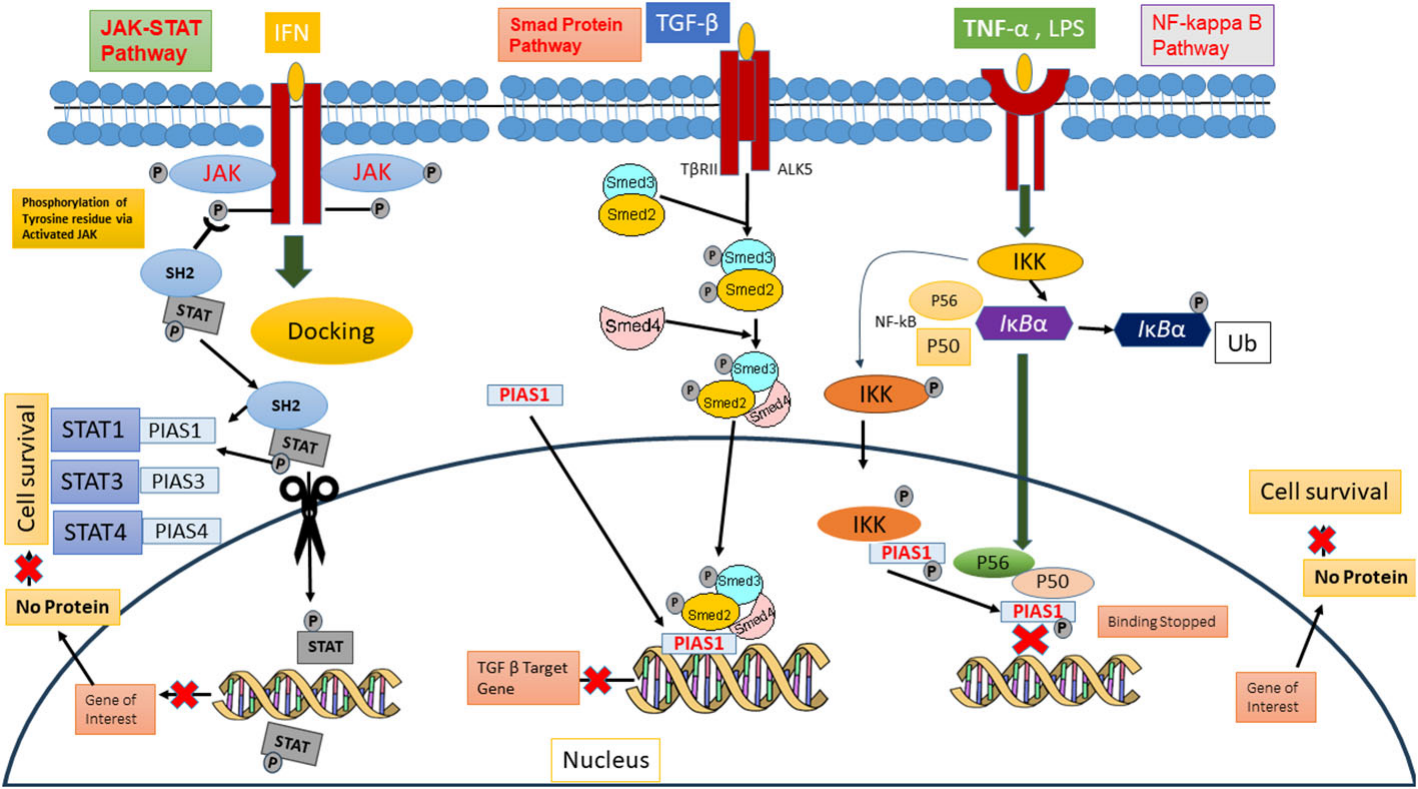
PIAS as downregulators of JAK/STAT, Smed signaling pathways, and NF kb pathway.

PIAS protein was first recognized to inhibit STAT in 1997 (26). Cytokines binding on cell surface receptor stimulates the Janus kinase, activators of transcription signal transduction pathways, and stress-activated/mitogen-activated protein kinase pathways that are responsible for various cellular responses (2). In mammals, seven members of the STAT family (STAT1-7) are conserved tyrosine residue, which are phosphorylated by JAKs. The term JAK derives from the Roman two-faced God having two domain categorizations, such as the catalytic domain and kinase-like domain. Upon binding of cytokine, its receptor activates the JAKs, ultimately causing trans-phosphorylation of cytoplasmic transcription factors (27). This phosphorylation resulting into dimer formation of specific STAT proteins is due to abandoned docking site that moves to the nucleus either to stimulate or suppress regulatory elements for gene transcription. Biochemical assays revealed that PIAS proteins block the DNA binding ability of STATs. PIAS1 interacts with the dimeric form of STAT1, while PIAS2x inhibits the transcriptional potential of STAT1 and STAT4. Interestingly, STAT3 losses its transcriptional activity because PIAS3 negatively regulates its DNA attaching ability present in homodimer or heterodimer form (27). The PIAS protein indeed functions as a transcriptional regulator not only in JAK/STAT pathway but also in other pathways such as NF-κB, p73, p53, and Smad proteins, by modulating their activity and down streaming the gene expression 37088348, 31758961, and 34054823. In the NF-κB pathway, PIAS is an important negative regulator that interacts with p56 subunit of NF-κB in the nucleus and blocks the DNA binding activity of p56 both *in vitro* and *in vivo* (28), although PIAS1 binds directly with DNA and stopped NF-mediated transcription. Inflammatory stimuli, such as TNF and LPS, activate the IKKa kinase, which translocate into the nucleus. Inside the nucleus, IKKa interacts with PIAS1, leading to the phosphorylation of PIAS1 at Ser90. The SUMO E3 ligase activity of PIAS1 is crucial for IKKa-mediated PIAS1 phosphorylation. Following phosphorylation, PIAS1 dissociates from IKKa and binds to the promoters of PIAS1-regulated genes, contributing to transcriptional repression (29). PIAS protein integrates signals from other signaling pathway to influence the NF-κB activity indirectly. These proteins also interact with p73, a member p53 tumor suppressor family, and play a significant role in its modulating activity through SUMOylation, altering its transcriptional regulator and protein–protein interaction, thereby influenced the cellular processes such as apoptosis and differentiation (5, 30). PIAS SUMOylates the p73 α and decreases the p73 transcriptional activity on several genes such as Bax and MDM in HEK293 cells at G1-to-S phase of cell cycle (31). Thus, PIAS strongly represses the transcriptional activity of p53 through promoting the apoptosis via upstreaming the Bax Level (32) (**Figure 3**).

## 5 The process of SUMOylation

SUMOylation and ubiquitination, two important components of ubiquitination proteasome system, have significant roles in protein homeostasis and signal transduction (33). Small ubiquitin-like modifier (SUMO) is colossal covalent change that combines with the target protein at lysine residue by recruiting enzymatic cascade (34). The modification by SUMO usually regulates protein–protein interactions through accumulation of binding partners that dock specific SUMO-interaction motifs (SIMs) (35). Mammals have four SUMO isoforms, which are SUMO 1–4 (36). SUMOylation regulates various biological processes including stabilization of protein structure, DNA damage repair, carcinoma, embryonic development, cell proliferation, immune responses, and apoptosis (37). Enzymatic cascade of SUMOylation comprised E1 (activator), E2 (conjugase), and E3 (ligase) enzymes (35). In humans, the SUMOylation process is triggered by sentrin-specific protease 1 (SENP) for proteolytic cleavage at carboxyl-terminal that exposes a terminal diglycine GG motif. The SUMO activation enzymes E1 and E2 form heterodimer (ATP dependent), composed of Aos1-Uba2 and SAE1-SAE2 proteins, respectively. To activate SUMO protein, the heterodimer Aos1/Uba2-mediated adenylation through ATP-dependent reaction stimulates the linkage of its carboxyl group with heterodimers SAE2/Uba2. E1 and E2 catalytic cysteine residues congregate via E2 enzyme Ubc9 to promote thioester transfer. However, the mechanism is still to be elucidated (38). SUMO E3 ligase plays a pivotal role in targeting protein; it binds with specific lysine residue in the substrate, and this conjugation can lead towards poly-SUMOylation (39). The modification of the SUMOylation process by removal of the SUMOglycine residue from the lysine substrate by the SUMO family of proteases indicates that SUMOylation is reversible (36).

### 5.1 SUMOylation and cancer

SUMOylation is a post-translational alteration that has arisen in recent decade as a mechanism involved in controlling several biological processes, which is essential in vertebrates (40). Dysregulation of SUMOlyation is linked with several age-related disorders and cancer formation (41). SUMO machinery is overexpressed in cancer cells to recruit or sustain tumorigenesis. E1-conjugated enzyme has been found overexpressed in colorectal cancer. A mechanistic investigation uncovered the catalytic subunit of E1 (SAE2), which reduced the tumor initiation (42).

Recent evidence revealed that the SUMOylation of Grb2 (growth factor receptor-bound protein 2) is crucial for the amplification of Ras/MEK/ERK cascade. Grb2 SUMOylation recruits Sos1 (son of sevenless homolog 1) for the formation of Grb2-Sos1 results in the initiation of signaling pathway RAS/MEK/MAPK that consequently plays pivotal roles in carcinogenesis, cell migration, and tumor development (43).

Mutation in regulatory gene such as MYC is involved in cell proliferation and tumorigenesis. Myc stimulates the SAE1 transcription, and research demonstrated that synthetic lethality of Myc was associated with silencing of SAE1/2 enzymatic activity. In cancer cells, downregulation of SAE1/2 and elevated level of Myc showed significant reduction in metastasis (36). Furthermore, the overexpression of SAE1/2 and Ubc9 has been found in hepatocellular carcinoma and pancreatic and breast cancer (44). The implication of SUMOylation in different cancers has already been described, but here, we will discuss PIAS/SUMO interaction with respect to cancer.

### 5.2 PIAS/SUMO interaction in cancer

The most frequently reported PIAS family member in cancer development are PIAS1 and PIAS3. These proteins perform as SUMO-specific ligases and trigger the SUMOylation of tumor suppressor p53 and protooncogene in non-small lung cell carcinoma. PIASy stops p53-mediated transactivation without affecting apoptosis ability (45). Contrastingly, SUMO E3 ligase PIAS3-Smurf2 SUMOylation pathway represses the breast cancer cell-derived organoids. PIAS3 reduction in breast tumor promotes the PIAS3 and Smurf2 pathway in tumor progression and metastasis; PIAS3-Smurf2 SUMOylation mechanism still requires more investigation in breast tumor metastasis (46). PIASxα is a novel ligase that reduces ubiquitation of PTEN to increase protein stability. PTEN regulates the cell cycle by blocking phosphatidylinositol 3-kinase (PI3K)-Akt signaling pathway (47). In addition, the overexpression of PIAS3xα deregulates the cyclin D kinase (CDK) that inhibits the cell proliferation (23). PIAS3xα deficiency may link with PTEN ubiquitation and overexpression of CDKs and cyclin D to promote the uncontrolled cell division. PIAS1 is a putative SUMO E3 ligase that extends breast cancer 1 (AIBI) half-life and is expressed excessively in approximately 60% of breast tumors. In breast tumor, AIBI modulates estrogen receptor α (ERα)-mediated gene expression. AIBI SUMOylation catalyzed by the E3 ligase inhibits AIB1 activity via reduced interaction with ERα. PIAS1-mediated AIBI SUMOylation needs to be further investigated for breast cancer therapy (48).

### 5.3 PIAS1 SUMOylation: cancer development and progression

**Figure 4:**
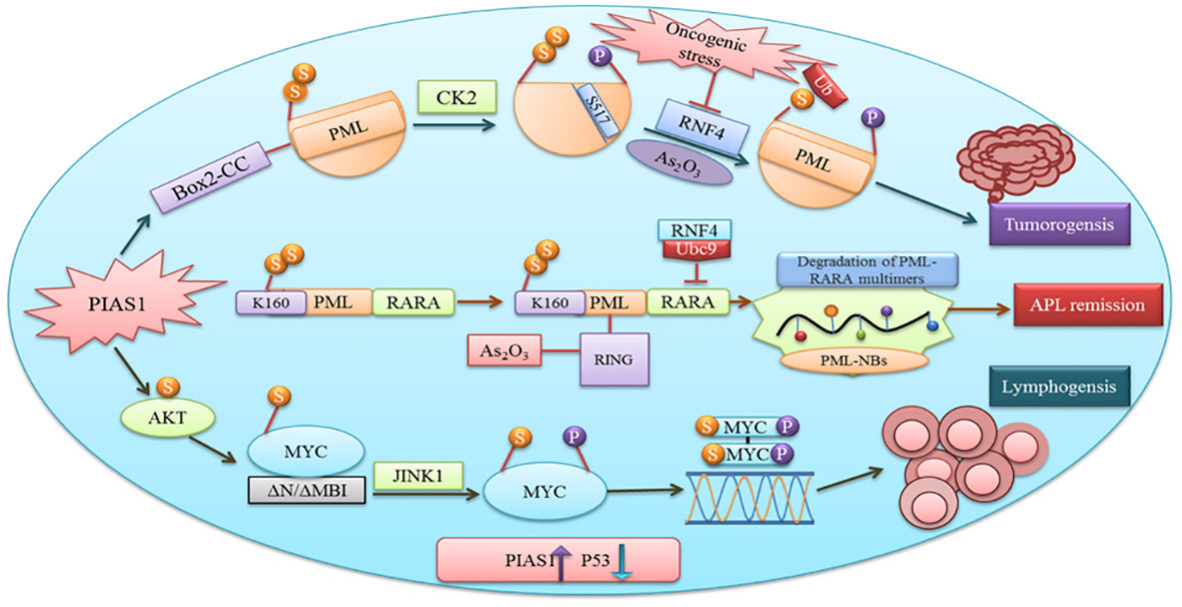
PIAS1 promotes tumorigenesis via SUMOylation and proteosomal degradation of PML. Also shown is the antagonistic role of PIAS1 in APL remission by recruiting RNF4-mediated ubiquitin degradation. Recruitment of JNK1 after PIAS1 mediated SUMOylation of MYC contributing to B-cell lymphomagenesis.

PIAS have a variety of functions including cell proliferation, differentiation, apoptosis, tumor development, and immune responses (2). Cancer proliferation, progression, and response to therapies depend upon the interaction between malignant cells and tumor microenvironment. Proteins involved in tumorigenesis rely on SUMOylation (49). PIAS1 and PIASxα co-localize with the resident protein of PML nuclear bodies that are renowned as SUMO1 and SUMO2. Box2-CC domain of PML facilitates its interaction with PIAS1 and PIASxα. Neither PIASxα nor point mutation at SUMOylation site affects the capability of PIAS1-mediated PML degradation (**Figure 4**). Casein kinase II (CK2) overexpression and physical interaction with other proteins play an important role in tumorigenesis. PIAS1 and CK2 interaction aberrantly leads to ubiquitin proteosomal degradation of tumor suppressor protein PML (50).

SUMOylation also regulates hexokinase 2 at K315 and K492 binding sites; thus, SUMO-defective hexokinase 2 preferably binds to mitochondria and also enhances glucose consumption and lactate production and downregulates mitochondrial respiration. This reprogramming supports prostate cancer cell proliferation and enhances the cells from chemotherapy-induced cell apoptosis (51). SUMOylation regulates YTHDF2 binding to the K571 site and promotes binding mRNA degradation and tumor progression via upstreaming of its binding affinity with m6A-modified mRNA (52). Monensin is an antibiotic that acts as anti-ovarian cancer by inhibiting the MEK-ERK pathway and also enhancing the MEK1 SUMOylation (53). In another study, SYNJ2BP-COX16 promoted breast cancer via phosphorylation with DRP1, mitochondrial fission, and SUMOylation at K107 residue. Thus, SYNJ2BP-COX16 is a novel target for treatment of breast cancer (54). Similarly, Ginkgolic acid is another therapeutic potential that inhibits the growth and invasion of many cancers via hindering the SUMOylation of IGF-1R (54).

MYC after binding with the DNA element CACGTG over the dimerization with MAX acts as a transcriptional activator (55). Gene translocation or amplification promotes MYC oncogenic activities in a number of human cancers. Findings recommend that SUMOylation is obligatory to tolerate aberrant MYC activation (56). Physical interaction between PIAS1 SUMO E3 ligase and MYC encouraged its carcinogenic activity in lymphomas. PIAS1 SUMO E3 ligase through MYC SUMOylation promotes its transcription activity, upregulates phosphorylated MYC at S62, and offers docking site for protein kinase JINK1 (57). An overexpression of PIAS1 and MYC was observed in stimulated B cells, substantial subset of prime B-cell lymphomas, and other cancer types. Moreover, the inhibition of SUMOylation persuades the apoptosis of MYC-dependent lymphoma cells and signifies it as an attractive therapeutic option (58).

The antagonistic role of specific PIAS1-mediated SUMOylation stabilizes PML-RARA necessary for its therapeutic action. Subsequent binding of the RNF4 ubiquitin E3-ligase to poly-SUMOylated PML-RARA resulted in proteasomal degradation and APL remission (50). Thus, PIAS1 is obligatory for PML-RARA degradation.

### 5.4 PIASY ligase as suppressor of Von Hippel–Lindau

PIAS4 also acts as specific E3-type SUMO ligase. Von Hippel–Lindau (VHL) plays a pivotal role in tumor suppression activity, which is lost upon SUMOylation. Deficiency of von Hippel–Lindau leads to constitutive initiation of hypoxia-inducible factor (HIF) along with HIF target gene expression causing tumors. Three family members are crucial for the regulation of hypoxia signaling pathway. Under hypoxia condition, HIF-1α translocates into the nucleus where it binds with PIAS4, which SUMOylates HIF1α within the C-terminal. SUMOylation enhances HIF1α-dependent transcription activity, attaches to VHL, and is degraded uniformly under hypoxia condition (59). An investigation revealed that PIAS4 stimulates HIF1α signaling upon the interaction and SUMOylation of VHL in pancreatic cancer and renal clear-cell carcinoma (60).

Synovial sarcoma is a soft tissue cancer in which oncoproteins like SYT-SSX1 and SYT-SSX2 are activated upon chromosomal translocation. A protein known as NOCOA3 is upregulated by SYT-SSX1, which leads to the development of many cancers. The interaction of SUMO E3 ligase PIAS4 with SYT-SSX1 increases the SUMOylation of its substrate NOCOA3 and its binding protein NEMO in synovial sarcoma. PIAS4-mediated SUMOylation is associated with upregulation of nuclear receptor coactivator 3, which triggers adverse effects in normal cells (61).

### 5.5 Smad and PIAS protein interaction promote cancer

SMADs are a class of proteins that deliver extracellular signals from the TGF-β ligand to the nucleus to control transcription (62). PIAS proteins were reported to modulate the transcriptional function of SMAD that arbitrate the TGF-β biological activities (63).

TGF-β plays as a key regulator for cancer progression and immune evasion. USP8 promotes the metastasis and its therapeutic advantage suppresses the metastatic activity 35811497. PRMT5 interacts with SMAD4 and the role of SMAD4 R361 methylation to control the TGF-β during the metastasis (64).

In various cancer cells, PIAS1 enhances the transcriptional function of the Smad2/Smad4 protein complex. TGF-β-activating R-Smad/Co-Smad complex promotes inhibition of cyclin-dependent kinase (CDK) to induce the cell growth arrest by direct activation of the promoter region of p21^WAF1/Cip1^ gene. PIAS1 is crucial for zinc-induced Smad4 pathway activation. Impairment in Smad pathway contributes to carcinogenesis due to the escape from growth inhibition (63), but how PIAS1 interacts with Smad still needs to be investigated.

PIAS3 is downregulated by Smad6 through the ubiquitin–proteasome pathway. PIAS3 interacts with Smad2, 3, 4, and 6 members of SMAD family. In the case of Smad6, MH2 domain and PIAS3 ring domain are responsible for their interaction that degrades PIAS3 and promotes STAT3 activity in glioblastoma. Another protein family member Smurf E3 ubiquitin–protein ligase catalytic domain interacts with the PY motif of Smad6 and facilitates PIAS3 degradation in order to promote tumor growth, invasion, and survival (65).

### 5.6 PIAS3 as a target of microRNAs in tumor

**Figure 5:**
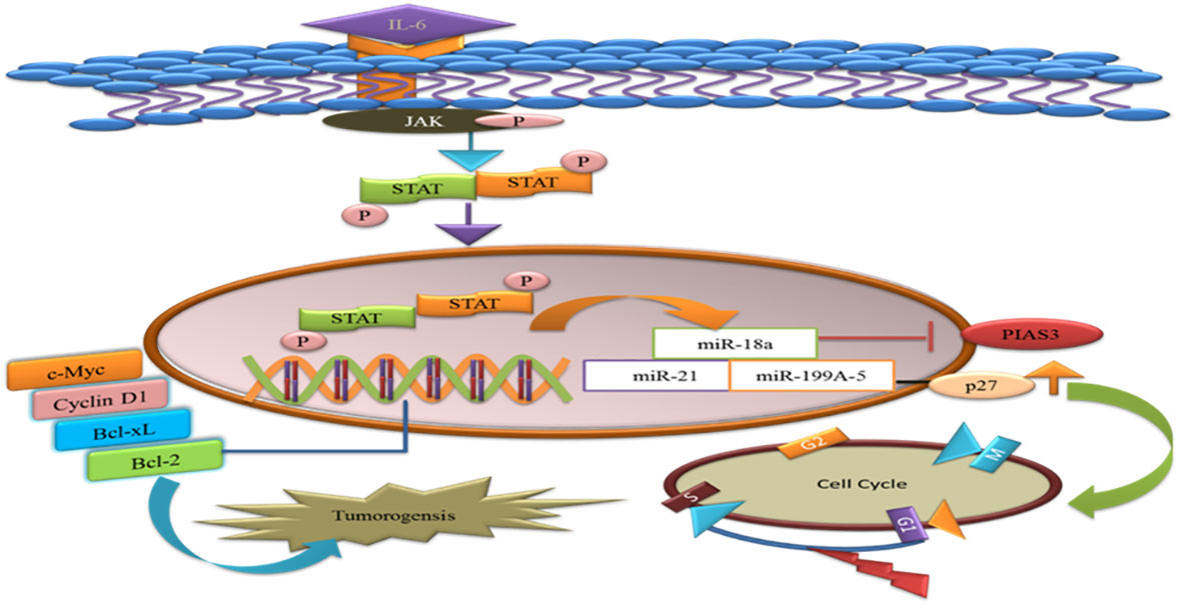
MicroRNAs target the PIAS3 and upregulate the STAT3 that promotes anti-apoptotic genes and tumorigenesis. p27 is targeted by miR-a99a-5 and delays G1/S phase of the cell cycles.

MicroRNAs are single-stranded RNAs that function as posttranscriptional modulators of gene expression. MiR-18a binds to the potential binding site 3′UTR of PIAS3. During gastric adeno-carcinogenesis, the upregulation of miR-18a miR-21 suppresses PIAS3 expressions. A recent work demonstrated that overexpression of the microRNA, miR-199a-5, negatively targets PIAS3 and p27 in osteosarcoma. The upregulation of miR-199a-5 enhanced the phosphorylation of STAT3 and ectopic overexpression of p27 that delays the G1-S phase change in the cell cycle. Thus, activated STAT3 helps tumor cells to avoid apoptosis and supports their proliferation (66). Therefore, introducing miRNA inhibitors will be the potential therapeutic approach valued for cancer therapy (**Figure 5**).

Tumor exosome-derived miR-9 and miR-181a activate the JAK/STAT pathway by targeting the PIAS3 and SOCS3, thus promoting the expression of eMDSCs and might be providing as novel target for IL-6^high^ breast cancer treatment (67). miR-199a-5p and microRNA-543 participate in cancer progression and proliferation via targeting the PIAS3 in cervical cancer and colorectal cancer (68, 69).

## 6 Modulated expression of PIAS proteins in different cancers

Recent research has demonstrated the relationship between aberrant expression of PIAS proteins and clinical pathological condition in several cancers.

### 6.1 PIAS1

The antagonistic expression of PIAS1 is involved in aberrant signaling pathways during carcinogenesis. Since PIAS proteins comprised SP-RING that exhibits SUMO E3 ligase activity, PIAS1-dependent SUMOylation was observed in the modulation of numerous oncogenes and tumor suppressors genes like *AKT*, *BRCA1*, and *BRCA2* (57). PIAS1 acts as a cell cycle regulator that catalyzes the SUMOylation of tumor suppressor p53 and p73 and promotes the cell proliferation (70). In lung cancer and osteosarcoma cells, PIAS1, primarily overexpressed throughout the S phase, may attach to and SUMOylate p73, thus hindering the transcriptional activity of p73 that is followed by a reduction in p21 (16). An overexpression of PIAS1 has been reported in lung cancer (71). PIAS1 gene contributes to nuclear accumulation of focal adhesion kinase FAK, where FAK accelerates p53 knockdown, ultimately promoting NSCLC progression (72). PIAS1 is overexpressed in EC; thus, the upregulation of miR-182-5p and miR-96-5p downregulates the PIAS level in EC (73).

### 6.2 PIAS2

PIAS2 gene expression is significantly reduced in cancerous tissues as compared to its adjacent non-cancerous tissues (24). A low level of PIASxα, a splice variant of PIAS2, is involved in tumorigenesis and cell proliferation in osteosarcoma tissues (23). PIAS2 interacts with UXT protein, an important co-regulator of transcription factors such as androgen receptor (AR), in the cytoplasm and nucleus of human cervical carcinoma (74). PIAS2 interacts with ZFHX3 and enhances its activity in cell proliferation in cancers cells (75).

### 6.3 PIAS3

PIAS3 is generally found in a number of human tissues such as the spleen, thymus, prostate, testis, ovary, colon, and peripheral blood. A domain structure PINIT found in PIAS3 can induce apoptosis by inhibiting the transcriptional activity of STAT3 (2). Various lines of evidence suggest that low expression of PIAS3 supports cancer cells proliferation or promotes tumorigenesis. PIAS3 mRNA expression is instantaneously silenced, which influences JAK/STAT signaling cascade in gastric, medulloblastoma, breast, and lung cancers (76). PIAS3 level is negatively associated with aberrant expression of STAT3 and its downstream targets such as survivin, Bcl-xL, and c-Myc in colorectal cancer (77). In lymphocytic leukemia, various aberrant signaling pathways in tumor microenvironment, including ZAP-70 protein, MAPK, and STAT3, stimulate the expression of post-translational regulator microRNA (miR-21) and other tumor suppressor genes (PTEN, PDCD4, and PIAS3). Upregulation of miR-21 expression with interleukin-4 (IL-4) promotes oncogenic processes through downregulation of tumor suppressor genes *PTEN*, *PDCD4*, and *PIAS3* (78). Another member of microRNA class is miR‐18a, which is negatively linked with PIAS3 expression in malignant mesothelioma (79). In glioblastoma, tri‐partite motif‐containing protein 8 (TRIM8) and nuclear-Smad6 arbitrate the ubiquitination that persuades degradation of PIAS3, which in turn promotes STAT3 activation (80).

### 6.4 PIAS4

**Figure 6:**
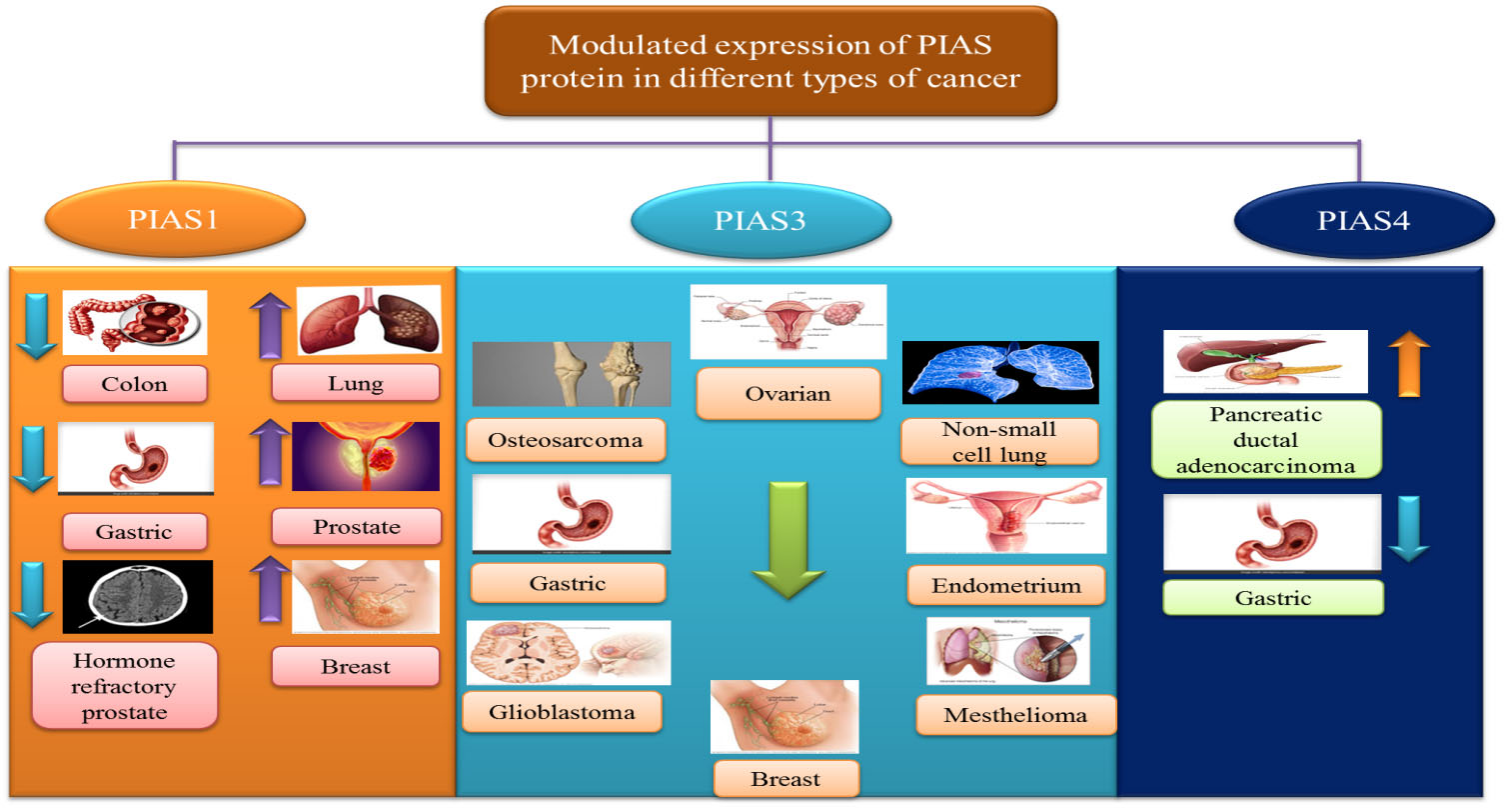
PIAS proteins promote cancer due to their controversial modulated expressions.

PIAS4 is widely expressed in the testis. It is the smallest protein among PIASs that encodes one splice variant PIAS4E6 (81). Upregulation of PIAS4 has been reported in human cancers. PIAS4 interacts and inhibits p53-mediated transactivation of its downregulators like Bax and p21 in NSCLC to inhibit apoptosis (60). PIAS4 with transcriptional co-repressor of androgen receptor interacts with DNA-binding domain of the AR that is essential for prostate cancer development and progression (**Figure 6**).

## 7 PIAS1 suppresses invasive growth of tumor via SnoN SUMOylation

PIAS proteins have the ability to obstruct caspase activity, neutralize the activation of pro-caspases, and act as ubiquitin ligases. PIAS protein is involved in the negative regulation of apoptosis through the proteosomal degradation of pro-apoptotic proteins and STAT, which has been reported in a number of cancers (82). PIAS1 and TIF1gamma promote the SnoN SUMOylation and suppression of epithelial mesenchymal transition cancers cells, but the regulation of EMT is still unclear (83).

**Figure 7:**
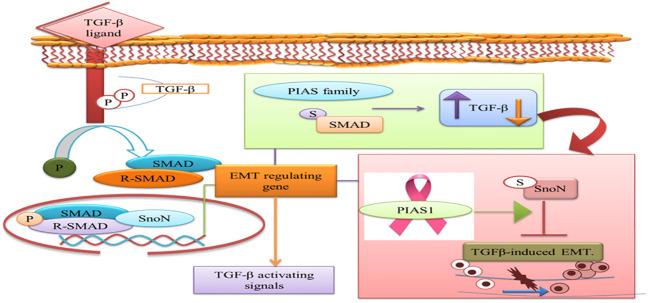
PIAS family either increases or decreases TGF-β signaling pathway that requires SMAD protein to translocate into the nucleus. PIAS1 modulated TGF-β-induced EMT complex through SUMOylation of SnoN. TGF-β, transforming growth factor beta; PIAS, protein inhibitor of activated STAT; EMT, epithelial–mesenchymal transition.

The prognostic value of PIAS1 revealed the detailed mechanism of the protein in SnoN SUMOylation, which supports great opportunity for breast cancer therapy (84). SnoN is a negative feedback inhibitor of transforming growth factor beta signaling (TGF-β). The biphasic role of TGF-β in advanced phases of cancer allows cells to metastasize through the induction of epithelial–mesenchymal transition (EMT) (85). During EMT, migration of cancer cells from the primary site of tumor and invasion at distant sites occur. TGF-β requires Smad- and MAD-related protein family members, Smad2 and Smad3, to transduce their signals to the nucleus. Thus, TGF-β-induced EMT pathway stimulates tumor progression later in epithelial tissues. SnoN and SUMO E3 ligase PIAS1 has emerged as an EMT regulator (86). In epithelial cells, PIAS1-SnoN SUMOylation inhibits TGF-β-induced EMT, which suggests the fundamental role of SnoN-SUMOylation in cancer progression. Foregoing investigation has revealed PIAS1 as a biomarker for breast cancer patients (85). The inhibition of TGF-β-induced EMT through SUMO E3 ligase PIAS1 and SnoN SUMOylation raises a question whether or not low level and subcellular localization of PIAS1 enhanced TGF-β-induced EMT and cancer metastasis (**Figure 7**).

## 8 Natural activators

**Figure 8:**
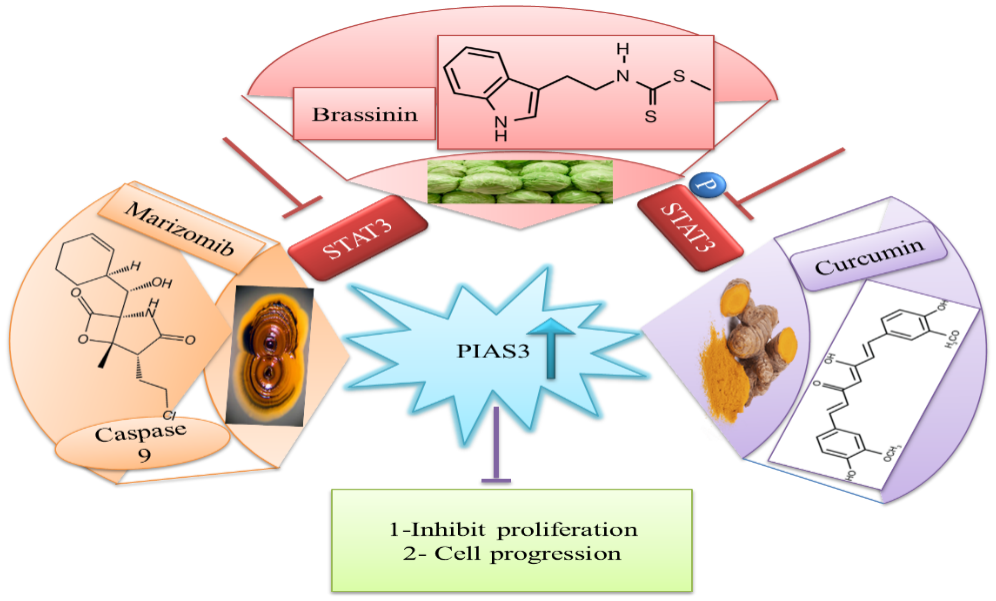
Natural activators of PIAS3 that negatively regulate STAT3 expressions. STAT3, signal transducer and activator of transcription 3; PIAS3, protein inhibitor of activated STAT3.

The downregulation of PIAS3 leads to the upregulation of STAT3, which promotes multiple oncogenic pathways; thus, it is an important target for cancer therapies (87). Considering these facts, few efforts have been done for the identification of natural activators to restore PIAS3 levels in cancer cells (88). Ascochlorin is an isoprenoid antibiotic obtained from phytopathogenic fungus *Ascochyta viciae.* Ascochlorin-induced inhibition of STAT3 substantially enhanced the expression of PIAS3 protein that significantly suppressed cancer growth in HepG2 cells. Another natural compound, Curcumin, enhances PIAS3 expressions and inhibits STAT3 phosphorylation in ovarian and endometrial cells in cancer (89). Brassinin (BSN), a phytoalexin, first isolated from cabbage, has anti‐tumor effects via enhancing PIAS3 expression. BSN inhibits the IL-6-induced STAT3 phosphorylation, which involves two inhibitors of STAT3, namely, PIAS3 and SOCS-3 (**Figure 8**). Paclitaxel is a semi-synthetic taxane obtained from the bark of the Pacific yew tree and is used as a therapeutic agent in NSCLC, but due to its severe side effects, potential combination of BSN and paclitaxel is used to diminish the lethality of chemotherapy during the treatment of lung cancer (90). Proteasomal inhibitors bortezomib or marizomib induce caspase 9 and increase PIAS3 expressions that initiated apoptosis and inhibit STAT3 activity in glioblastoma cells (91).

## 9 Clinical applications of PIAS in cancer

PIAS proteins are expressed in different cancer; thus, targeting PIAS might be a novel approach for the treatment of cancer. Samples from 25 breast cancer patients and non-cancerous tissues from the same breast were obtained and PIAS3 mRNA and quantified. PIAS3 mRNA expression was considerably lower in these patients as compared to non-cancerous tissues; thus, PIAS3 mRNA might be crucial in breast cancer development (92).

PIAS functions as a suppressor within the STAT3 signaling pathway, a critical regulator of cellular processes such as proliferation, differentiation, and apoptosis. Through its regulatory influence on the STAT3 pathway, PIAS emerges as a potential therapeutic target for addressing STAT3-dependent cancers. Manipulating the STAT3 pathway with PIAS holds promise for the treatment of cancers driven by aberrant STAT3 activity (58).

PIAS1 expression caused by Ad5/F35 virus in gastric cancer patients decreased the cell proliferation and invasion. These results indicate that PIAS1 might perform as a cancer suppressor to control metastasis (93). The expression of PIASxα from tissue samples of 29 osteosarcoma patients was analyzed utilizing reverse transcription–quantitative polymerase chain reaction and Western blot. The outcomes showed reduced expression of PIAsxα in these patients. Moreover, succeeding the overexpression of PIAsxα, the apoptosis was considerably increased. Increased expression of PIAsxα decreased tumor formation in the mouse model (94).

miR-182-5p and miR-96-5p inhibitors show the ability to increase PIAS1 expression in endometrial cancer (EC) cells. This increase in PIAS1 levels, in turn, plays a key role in inhibiting STAT3 activity. Furthermore, treatment with ectopic expression of PIAS1 and STAT3 inhibitors further suppresses STAT3 activity and decreases miR-182-5p and miR-96-5p levels in EC cells. Therefore, these results suggest that inhibitors act to disrupt the negative feedback regulatory loop between PIAS1 and STAT3, offering a promising approach for EC management by interfering with this molecular pathway (73). Quantitative SUMO proteomics analysis, following CRISPR/Cas9 knockout of individual PIAS genes elucidates novel insights into the regulatory roles of PIAS SUMO E3 ligases, shedding light on both specific and overlapping mechanisms governing cell proliferation and the cell cycle (95).

Curcumin is a dihydroxyphenolic compound possessing anti-cancer activity in many tumors. Normal ovarian and endometrial cells showed increased expression of PIAS3, while in tumor cells, the expression was considerably decreased. Curcumin enhanced PIAS3 expression in tumor cells. In a nutshell, curcumin inhibits JAK-STAT signaling through activation of PIAS3, thus decreasing STAT3 phosphorylation and cancer cell growth (89).

Brassinin is a phytoalexin and has been documented to have anticarcinogenic, chemopreventive, and antiproliferative potential (96–99). Brassinin prevents STAT3 signaling by modulating PIAS3, leading to reduced cancer growth (b). In short targeting PIAS by its inhibitors in the future could be a promising therapeutic strategy to decrease the cell proliferation and treat cancer (90).

## 10 Conclusion and future challenges

Recent evidence indicated that the antagonistic role of PIAS members as SUMO E3 ligases is emerging as positive regulator of intricate oncogenic networks. Recent reports suggest that PIAS protein members are differently expressed in various cancer types. Therefore, targeting PIAS proteins and downstream targets is a novel treatment approach for tumor therapy. To date, various investigations have focused on the development of pharmacological or natural inhibitors and activators regarding their expressions. However, more work should be concentrating on the molecular mechanism of natural activators of PIAS3. The interaction of PIAS1 and PIAS2 with SMAD, and PIAS1, PIAS2, and PIAS3 with Von Hippel–Lindau should be investigated in future studies. A detailed study on possible mechanism between miRNAs and PIAS3 interactions should be carried out. The overall expression of PIAS2 and PIAS4 in tumor should be described more in detail. Further studies on screening the drugs to stabilize antagonistic PIAS protein expressions offer potential therapeutic index for cancer treatment.

## Author contributions

XL: Writing – review & editing, Funding acquisition, Conceptualization. AR: Writing – review & editing, Validation, Supervision. FS: Writing – original draft. MH: Writing – review & editing, Writing – original draft, Visualization.

## References

[1] Rytinki MM Kaikkonen S Pehkonen P Jaaskelainen T Palvimo JJ. PIAS proteins: pleiotropic interactors associated with SUMO. Cell Mol Life Sci. (2009) 66:3029–41. doi: 10.1007/s00018-009-0061-z PMC11115825 19526197

[2] Niu GJ Xu JD Yuan WJ Sun JJ Yang MC He ZH. Protein inhibitor of activated STAT (PIAS) negatively regulates the JAK/STAT pathway by inhibiting STAT phosphorylation and translocation. Front Immunol. (2018) 9:2392. doi: 10.3389/fimmu.2018.02392 30416501 PMC6212522

[3] Liu B Liao J Rao X Kushner SA Chung CD Chang DD. Inhibition of Stat1-mediated gene activation by PIAS1. Proc Natl Acad Sci U.S.A. (1998) 95:10626–31. doi: 10.1073/pnas.95.18.10626 PMC27945 9724754

[4] Murphy JM Tannahill GM Hilton DJ Greenhalgh CJ. The negative regulation of JAK/STAT signaling. In: Handbook of cell signaling. Academic Press. Elsevier (2010). p. 467–80. doi: 10.1016/B978-0-12-374145-5.00064-4

[5] Wu M Song D Li H Yang Y Ma X Deng S. Negative regulators of STAT3 signaling pathway in cancers. Cancer Manag Res. (2019) 11:4957–69. doi: 10.2147/CMAR PMC6549392 31213912

[6] Chung CD Liao J Liu B Rao X Jay P Berta P. Specific inhibition of Stat3 signal transduction by PIAS3. Science. (1997) 278:1803–5. doi: 10.1126/science.278.5344.1803 9388184

[7] Starr R Hilton DJ. Negative regulation of the JAK/STAT pathway. Bioessays. (1999) 21:47–52. doi: 10.1002/(SICI)1521-1878(199901)21:11.0.CO;2-4 10070253

[8] Tan J Hall SH Hamil KG Grossman G Petrusz P Liao J. Protein inhibitor of activated STAT-1 (signal transducer and activator of transcription-1) is a nuclear receptor coregulator expressed in human testis. Mol Endocrinol. (2000) 14:14–26. doi: 10.1210/mend.14.1.0408 10628744

[9] Junicho A Matsuda T Yamamoto T Kishi H Korkmaz K Saatcioglu F. Protein inhibitor of activated STAT3 regulates androgen receptor signaling in prostate carcinoma cells. Biochem Biophys Res Commun. (2000) 278:9–13. doi: 10.1006/bbrc.2000.3753 11071847

[10] Gross M Liu B Tan J French FS Carey M Shuai K. Distinct effects of PIAS proteins on androgen-mediated gene activation in prostate cancer cells. Oncogene. (2001) 20:3880–7. doi: 10.1038/sj.onc.1204489 11439351

[11] Sachdev S Bruhn L Sieber H Pichler A Melchior F Grosschedl R. PIASy, a nuclear matrix-associated SUMO E3 ligase, represses LEF1 activity by sequestration into nuclear bodies. Genes Dev. (2001) 15:3088–103. doi: 10.1101/gad.944801 PMC312834 11731474

[12] Duval D Duval G Kedinger C Poch O Boeuf H. The 'PINIT' motif, of a newly identified conserved domain of the PIAS protein family, is essential for nuclear retention of PIAS3L. FEBS Lett. (2003) 554:111–8. doi: 10.1016/S0014-5793(03)01116-5 14596924

[13] Long J Wang G Matsuura I He D Liu F. Activation of Smad transcriptional activity by protein inhibitor of activated STAT3 (PIAS3). Proc Natl Acad Sci U.S.A. (2004) 101:99–104. doi: 10.1073/pnas.0307598100 14691252 PMC314145

[14] Ehrmann J Strakova N Vrzalikova K Hezova R Kolar Z. Expression of STATs and their inhibitors SOCS and PIAS in brain tumors. *In vitro* and in *vivo* study. Neoplasma. (2008) 55:482–7. 18999875

[15] Sundvall M Korhonen A Vaparanta K Anckar J Halkilahti K Salah Z. Protein inhibitor of activated STAT3 (PIAS3) protein promotes SUMOylation and nuclear sequestration of the intracellular domain of ErbB4 protein. J Biol Chem. (2012) 287:23216–26. doi: 10.1074/jbc.M111.335927 PMC3391121 22584572

[16] Hoefer J Schafer G Klocker H Erb HH Mills IG Hengst L. PIAS1 is increased in human prostate cancer and enhances proliferation through inhibition of p21. Am J Pathol. (2012) 180:2097–107. doi: 10.1016/j.ajpath.2012.01.026 22449952

[17] Ghaffari S. PIAS adds methyl-bias to HSC-differentiation. EMBO J. (2014) 33:93–5. doi: 10.1002/embj.v33.2 PMC3989602 24421322

[18] Gur I Fujiwara K Hasegawa K Yoshikawa K. Necdin promotes ubiquitin-dependent degradation of PIAS1 SUMO E3 ligase. PloS One. (2014) 9:e99503. doi: 10.1371/journal.pone.0099503 24911587 PMC4049815

[19] Brown JR Conn KL Wasson P Charman M Tong L Grant K. SUMO ligase protein inhibitor of activated STAT1 (PIAS1) is a constituent promyelocytic leukemia nuclear body protein that contributes to the intrinsic antiviral immune response to herpes simplex virus 1. J Virol. (2016) 90:5939–52. doi: 10.1128/JVI.00426-16 PMC4907222 27099310

[20] Conn KL Wasson P Mcfarlane S Tong L Brown JR Grant KG. Novel role for protein inhibitor of activated STAT 4 (PIAS4) in the restriction of herpes simplex virus 1 by the cellular intrinsic antiviral immune response. J Virol. (2016) 90:4807–26. doi: 10.1128/JVI.03055-15 PMC4836348 26937035

[21] Lao M Shi M Zou Y Huang M Ye Y Qiu Q. Protein inhibitor of activated STAT3 regulates migration, invasion, and activation of fibroblast-like synoviocytes in rheumatoid arthritis. J Immunol. (2016) 196:596–606. doi: 10.4049/jimmunol.1403254 26667168

[22] Nakagawa K Kohara T Uehata Y Miyakawa Y Sato-Ueshima M Okubo N. PIAS3 enhances the transcriptional activity of HIF-1alpha by increasing its protein stability. Biochem Biophys Res Commun. (2016) 469:470–6. doi: 10.1016/j.bbrc.2015.12.047 26697750

[23] Wang J Ni J Yi S Song D Ding M. Protein inhibitor of activated STAT xalpha depresses cyclin D and cyclin D kinase, and contributes to the inhibition of osteosarcoma cell progression. Mol Med Rep. (2016) 13:1645–52. doi: 10.3892/mmr.2015.4705 26708148

[24] Taheri M Oskooei VK Ghafouri-Fard S. Protein inhibitor of activated STAT genes are differentially expressed in breast tumor tissues. Per Med. (2019) 16:277–85. doi: 10.2217/pme-2018-0070 31244388

[25] Aravind L Koonin EV. SAP - a putative DNA-binding motif involved in chromosomal organization. Trends Biochem Sci. (2000) 25:112–4. doi: 10.1016/S0968-0004(99)01537-6 10694879

[26] Zhang S Li C Wang W Wang C Sun C Chan S. Functional characterization of a protein inhibitor of activated STAT (PIAS) gene in Litopenaeus vannamei. Fish Shellfish Immunol. (2019) 94:417–26. doi: 10.1016/j.fsi.2019.09.007 31491531

[27] Seif F Khoshmirsafa M Aazami H Mohsenzadegan M Sedighi G Bahar M. The role of JAK-STAT signaling pathway and its regulators in the fate of T helper cells. Cell Commun Signal. (2017) 15:23. doi: 10.1186/s12964-017-0177-y 28637459 PMC5480189

[28] Liu B Yang R Wong KA Getman C Stein N Teitell MA. Negative regulation of NF-kappaB signaling by PIAS1. Mol Cell Biol. (2005) 25:1113–23. doi: 10.1128/MCB.25.3.1113-1123.2005 PMC544018 15657437

[29] Liu B Shuai K. Targeting the PIAS1 SUMO ligase pathway to control inflammation. Trends in pharmacological sciences (2008) 29:505–9. doi: 10.1016/j.tips.2008.07.008 PMC2701905 18755518

[30] Ghafouri-Fard S Hussen BM Nicknafs F Nazer N Sayad A Taheri M. Expression analysis of protein inhibitor of activated STAT in inflammatory demyelinating polyradiculoneuropathy. Front Immunol. (2021) 12:659038. doi: 10.3389/fimmu.2021.659038 34054823 PMC8149797

[31] Omran Z H.D. M Abdullah O Kaleem M Hosawi S A.a.-A. F. Targeting post-translational modifications of the p73 protein: A promising therapeutic strategy for tumors. Cancers (Basel). (2021) 13(8):1916. doi: 10.3390/cancers13081916 33921128 PMC8071514

[32] Schmidt D Müller S. Members of the PIAS family act as SUMO ligases for c-Jun and p53 and repress p53 activity. Proc Natl Acad Sci U.S.A. (2002) 99:2872–7. doi: 10.1073/pnas.052559499 PMC122440 11867732

[33] Dai X Zhang T Hua D. Ubiquitination and SUMOylation: protein homeostasis control over cancer. Epigenomics. (2022) 14:43–58. doi: 10.2217/epi-2021-0371 34875856

[34] Zhao X. SUMO-mediated regulation of nuclear functions and signaling processes. Mol Cell. (2018) 71:409–18. doi: 10.1016/j.molcel.2018.07.027 PMC6095470 30075142

[35] Nayak A Muller S. SUMO-specific proteases/isopeptidases: SENPs and beyond. Genome Biol. (2014) 15:422. doi: 10.1186/s13059-014-0422-2 25315341 PMC4281951

[36] Han ZJ Feng YH Gu BH Li YM Chen H. The post-translational modification, SUMOylation, and cancer (Review). Int J Oncol. (2018) 52:1081–94. doi: 10.3892/ijo PMC5843405 29484374

[37] Bettermann K Benesch M Weis S Haybaeck J. SUMOylation in carcinogenesis. Cancer Lett. (2012) 316:113–25. doi: 10.1016/j.canlet.2011.10.036 22138131

[38] Pichler A Fatouros C Lee H Eisenhardt N. SUMO conjugation - a mechanistic view. Biomol Concepts. (2017) 8:13–36. doi: 10.1515/bmc-2016-0030 28284030

[39] Sriramachandran AM Dohmen RJ. SUMO-targeted ubiquitin ligases. Biochim Biophys Acta. (2014) 1843:75–85. doi: 10.1016/j.bbamcr.2013.08.022 24018209

[40] Lara-Ureña N Jafari V García-Domínguez M. Cancer-associated dysregulation of sumo regulators: proteases and ligases. Int J Mol Sci. (2022) 23(14):8012. doi: 10.3390/ijms23148012 35887358 PMC9316396

[41] Sun Q Qing W Qi R Zou M Gong L Liu Y. Inhibition of sumoylation alleviates oxidative stress-induced retinal pigment epithelial cell senescence and represses proinflammatory gene expression. Curr Mol Med. (2018) 18:575–83. doi: 10.2174/1566524019666190107154250 30621561

[42] Du L Li YJ Fakih M Wiatrek RL Duldulao M Chen Z. Role of SUMO activating enzyme in cancer stem cell maintenance and self-renewal. Nat Commun. (2016) 7:12326. doi: 10.1038/ncomms12326 27465491 PMC4974481

[43] Qu Y Chen Q Lai X Zhu C Chen C Zhao X. SUMOylation of Grb2 enhances the ERK activity by increasing its binding with Sos1. Mol Cancer. (2014) 13:95. doi: 10.1186/1476-4598-13-95 24775912 PMC4021559

[44] van Doorn BA van der Does E Lubsen J Rijsterborgh H. [Reliability of blood pressure measurements; comparison of an electronic meter and a mercury manometer in family practice]. Ned Tijdschr Geneeskd. (1990) 134:1646–50. 2215707

[45] Nelson V Davis GE Maxwell SA. A putative protein inhibitor of activated STAT (PIASy) interacts with p53 and inhibits p53-mediated transactivation but not apoptosis. Apoptosis. (2001) 6:221–34. doi: 10.1023/A:1011392811628 11388671

[46] Chandhoke AS Chanda A Karve K Deng L Bonni S. The PIAS3-Smurf2 sumoylation pathway suppresses breast cancer organoid invasiveness. Oncotarget. (2017) 8:21001–14. doi: 10.18632/oncotarget.v8i13 PMC5400561 28423498

[47] Wang W Chen Y Wang S Hu N Cao Z Wang W. PIASxalpha ligase enhances SUMO1 modification of PTEN protein as a SUMO E3 ligase. J Biol Chem. (2014) 289:3217–30. doi: 10.1074/jbc.M113.508515 PMC3916526 24344134

[48] De Silva DD Rapior S Hyde KD Bahkali AH. Medicinal mushrooms in prevention and control of diabetes mellitus. Fungal diversity (2012) 56:1–29. doi: 10.1007/s13225-012-0187-4

[49] Gu Y Fang Y Wu X Xu T Hu T Xu Y. The emerging roles of SUMOylation in the tumor microenvironment and therapeutic implications. Exp Hematol Oncol. (2023) 12:58. doi: 10.1186/s40164-023-00420-3 37415251 PMC10324244

[50] Rabellino A Carter B Konstantinidou G Wu SY Rimessi A Byers LA. The SUMO E3-ligase PIAS1 regulates the tumor suppressor PML and its oncogenic counterpart PML-RARA. Cancer Res. (2012) 72:2275–84. doi: 10.1158/0008-5472.CAN-11-3159 PMC3342450 22406621

[51] Shangguan X He J Ma Z Zhang W Ji Y Shen K. SUMOylation controls the binding of hexokinase 2 to mitochondria and protects against prostate cancer tumorigenesis. Nat Commun. (2021) 12:1812. doi: 10.1038/s41467-021-22163-7 33753739 PMC7985146

[52] Hou G Zhao X Li L Yang Q Liu X Huang C. SUMOylation of YTHDF2 promotes mRNA degradation and cancer progression by increasing its binding affinity with m6A-modified mRNAs. Nucleic Acids Res. (2021) 49:2859–77. doi: 10.1093/nar/gkab065 PMC7969013 33577677

[53] Yao S Wang W Zhou B Cui X Yang H Zhang S. Monensin suppresses cell proliferation and invasion in ovarian cancer by enhancing MEK1 SUMOylation. Exp Ther Med. (2021) 22:1390. doi: 10.3892/etm 34650638 PMC8506924

[54] Wang M Wei R Li G Bi HL Jia Z Zhang M. SUMOylation of SYNJ2BP-COX16 promotes breast cancer progression through DRP1-mediated mitochondrial fission. Cancer Lett. (2022) 547:215871. doi: 10.1016/j.canlet.2022.215871 35998797

[55] Amati B. Myc degradation: dancing with ubiquitin ligases. Proc Natl Acad Sci U.S.A. (2004) 101:8843–4. doi: 10.1073/pnas.0403046101 PMC428433 15187232

[56] Kessler JD Kahle KT Sun T Meerbrey KL Schlabach MR Schmitt EM. A SUMOylation-dependent transcriptional subprogram is required for Myc-driven tumorigenesis. Science. (2012) 335:348–53. doi: 10.1126/science.1212728 PMC4059214 22157079

[57] Rabellino A Melegari M Tompkins VS Chen W Van Ness BG Teruya-Feldstein J. PIAS1 promotes lymphomagenesis through MYC upregulation. Cell Rep. (2016) 15:2266–78. doi: 10.1016/j.celrep.2016.05.015 PMC4899214 27239040

[58] Rabellino A Andreani C Scaglioni PP. The role of PIAS SUMO E3-ligases in cancer. Cancer research (2017) 77:1542–7. doi: 10.1158/0008-5472.CAN-16-2958 PMC5380518 28330929

[59] Kang X Li J Zou Y Yi J Zhang H Cao M. PIASy stimulates HIF1alpha SUMOylation and negatively regulates HIF1alpha activity in response to hypoxia. Oncogene. (2010) 29:5568–78. doi: 10.1038/onc.2010.297 20661221

[60] Chien W Lee KL Ding LW Wuensche P Kato H Doan NB. PIAS4 is an activator of hypoxia signalling via VHL suppression during growth of pancreatic cancer cells. Br J Cancer. (2013) 109:1795–804. doi: 10.1038/bjc.2013.531 PMC3790182 24002598

[61] Sun Y Perera J Rubin BP Huang J. SYT-SSX1 (synovial sarcoma translocated) regulates PIASy ligase activity to cause overexpression of NCOA3 protein. J Biol Chem. (2011) 286:18623–32. doi: 10.1074/jbc.M110.176693 PMC3099678 21454665

[62] Derynck R Zhang Y Feng XH. Smads: transcriptional activators of TGF-beta responses. Cell. (1998) 95:737–40. doi: 10.1016/S0092-8674(00)81696-7 9865691

[63] Brantley EC Nabors LB Gillespie GY Choi YH Palmer CA Harrison K. Loss of protein inhibitors of activated STAT-3 expression in glioblastoma multiforme tumors: implications for STAT-3 activation and gene expression. Clin Cancer Res. (2008) 14:4694–704. doi: 10.1158/1078-0432.CCR-08-0618 PMC3886729 18676737

[64] Liu A Yu C Qiu C Wu Q Huang C Li X. PRMT5 methylating SMAD4 activates TGF-β signaling and promotes colorectal cancer metastasis. Oncogene. (2023) 42:1572–84. doi: 10.1038/s41388-023-02674-x 36991117

[65] Jiao J Zhang R Li Z Yin Y Fang X Ding X. Nuclear Smad6 promotes gliomagenesis by negatively regulating PIAS3-mediated STAT3 inhibition. Nat Commun. (2018) 9:2504. doi: 10.1038/s41467-018-04936-9 29950561 PMC6021382

[66] Poliseno L Salmena L Riccardi L Fornari A Song MS Hobbs RM. Identification of the miR-106b~25 microRNA cluster as a proto-oncogenic PTEN-targeting intron that cooperates with its host gene MCM7 in transformation. Sci Signal. (2010) 3:ra29. doi: 10.1126/scisignal.2000594 20388916 PMC2982149

[67] Jiang M Zhang W Zhang R Liu P Ye Y Yu W. Cancer exosome-derived miR-9 and miR-181a promote the development of early-stage MDSCs via interfering with SOCS3 and PIAS3 respectively in breast cancer. Oncogene. (2020) 39:4681–94. doi: 10.1038/s41388-020-1322-4 32398867

[68] Qu D Yang Y Huang X. miR-199a-5p promotes proliferation and metastasis and epithelial-mesenchymal transition through targeting PIAS3 in cervical carcinoma. J Cell Biochem. (2019) 120:13562–72. doi: 10.1002/jcb.28631 30937952

[69] Su DW Li X Chen J Dou J Fang GE Luo CJ. MiR-543 inhibits proliferation and metastasis of human colorectal cancer cells by targeting PLAS3. Eur Rev Med Pharmacol Sci. (2020) 24:8812–21. doi: 10.1002/jcb.28631 32964969

[70] Puhr M Hoefer J Eigentler A Dietrich D Van Leenders G Uhl B. PIAS1 is a determinant of poor survival and acts as a positive feedback regulator of AR signaling through enhanced AR stabilization in prostate cancer. Oncogene. (2016) 35:2322–32. doi: 10.1038/onc.2015.292 PMC4865476 26257066

[71] Constanzo JD Tang KJ Rindhe S Melegari M Liu H Tang X. PIAS1-FAK interaction promotes the survival and progression of non-small cell lung cancer. Neoplasia. (2016) 18:282–93. doi: 10.1016/j.neo.2016.03.003 PMC4887597 27237320

[72] Yin H Yu Y. Identification of the targets of hematoporphyrin derivative in lung adenocarcinoma using integrated network analysis. Biol Res. (2019) 52:4. doi: 10.1186/s40659-019-0213-z 30717818 PMC6360726

[73] Xiao Y Huang W Huang H Wang L Wang M Zhang T. miR-182-5p and miR-96-5p target PIAS1 and mediate the negative feedback regulatory loop between PIAS1 and STAT3 in endometrial cancer. DNA and Cell Biology (2021) 40:618–28. doi: 10.1089/dna.2020.6379 33751900

[74] Kong X Ma S Guo J Ma Y Hu Y Wang J. Ubiquitously expressed transcript is a novel interacting protein of protein inhibitor of activated signal transducer and activator of transcription 2. Mol Med Rep. (2015) 11:2443–8. doi: 10.3892/mmr.2014.3023 PMC4337631 25434787

[75] Wu R Fang J Liu M A J Liu J Chen W. SUMOylation of the transcription factor ZFHX3 at Lys-2806 requires SAE1, UBC9, and PIAS2 and enhances its stability and function in cell proliferation. J Biol Chem. (2020) 295:6741–53. doi: 10.1074/jbc.RA119.012338 PMC7212658 32249212

[76] Li C Li H Zhang P Yu LJ Huang TM Song X. SHP2, SOCS3 and PIAS3 expression patterns in medulloblastomas: relevance to STAT3 activation and resveratrol-suppressed STAT3 signaling. Nutrients. (2016) 9(1):3. doi: 10.3390/nu9010003 28035977 PMC5295047

[77] Li H Gao H Bijukchhe SM Wang Y Li T. PIAS3 may represent a potential biomarker for diagnosis and therapeutic of human colorectal cancer. Med Hypotheses. (2013) 81:1151–4. doi: 10.1016/j.mehy.2013.09.022 24120699

[78] Carabia J Carpio C Abrisqueta P Jimenez I Purroy N Calpe E. Microenvironment regulates the expression of miR-21 and tumor suppressor genes PTEN, PIAS3 and PDCD4 through ZAP-70 in chronic lymphocytic leukemia. Sci Rep. (2017) 7:12262. doi: 10.1038/s41598-017-12135-7 28947822 PMC5612928

[79] He T Mccoll K Sakre N Chen Y Wildey G Dowlati A. Post-transcriptional regulation of PIAS3 expression by miR-18a in Malignant mesothelioma. Mol Oncol. (2018) 12:2124–35. doi: 10.1002/1878-0261.12386 PMC6275277 30259640

[80] Zhang C Mukherjee S Tucker-Burden C Ross JL Chau MJ Kong J. TRIM8 regulates stemness in glioblastoma through PIAS3-STAT3. Mol Oncol. (2017) 11:280–94. doi: 10.1002/1878-0261.12034 PMC5332279 28100038

[81] Schmidt D Muller S. PIAS/SUMO: new partners in transcriptional regulation. Cell Mol Life Sci. (2003) 60:2561–74. doi: 10.1007/s00018-003-3129-1 PMC11138616 14685683

[82] Malemud CJ. Negative regulators of JAK/STAT signaling in rheumatoid arthritis and osteoarthritis. Int J Mol Sci. (2017) 18(3):484. doi: 10.3390/ijms18030484 28245561 PMC5372500

[83] Chanda A Ikeuchi Y Karve K Sarkar A Chandhoke AS Deng L. PIAS1 and TIF1γ collaborate to promote SnoN SUMOylation and suppression of epithelial-mesenchymal transition. Cell Death Differ. (2021) 28:267–82. doi: 10.1038/s41418-020-0599-8 PMC7853041 32770107

[84] Dadakhujaev S Salazar-Arcila C Netherton SJ Chandhoke AS Singla AK Jirik FR. A novel role for the SUMO E3 ligase PIAS1 in cancer metastasis. Oncoscience. (2014) 1:229–40. doi: 10.18632/oncoscience.v1i3 PMC4278292 25594015

[85] Chanda A Chan A Deng L Kornaga EN Enwere EK Morris DG. Identification of the SUMO E3 ligase PIAS1 as a potential survival biomarker in breast cancer. PloS One. (2017) 12:e0177639. doi: 10.1371/journal.pone.0177639 28493978 PMC5426774

[86] Bonni S Bonni A. SnoN signaling in proliferating cells and postmitotic neurons. FEBS Lett. (2012) 586:1977–83. doi: 10.1016/j.febslet.2012.02.048 PMC3383335 22710173

[87] Dabir S Kluge A Mccoll K Liu Y Lam M Halmos B. PIAS3 activates the intrinsic apoptotic pathway in non-small cell lung cancer cells independent of p53 status. Int J Cancer. (2014) 134:1045–54. doi: 10.1002/ijc.28448 PMC4199747 23959540

[88] Dai X Ahn KS Kim C Siveen KS Ong TH Shanmugam MK. Ascochlorin, an isoprenoid antibiotic inhibits growth and invasion of hepatocellular carcinoma by targeting STAT3 signaling cascade through the induction of PIAS3. Mol Oncol. (2015) 9:818–33. doi: 10.1016/j.molonc.2014.12.008 PMC5528777 25624051

[89] Saydmohammed M Joseph D Syed V. Curcumin suppresses constitutive activation of STAT-3 by up-regulating protein inhibitor of activated STAT-3 (PIAS-3) in ovarian and endometrial cancer cells. J Cell Biochem. (2010) 110:447–56. doi: 10.1002/jcb.22558 20235152

[90] Lee JH Kim C Sethi G Ahn KS. Brassinin inhibits STAT3 signaling pathway through modulation of PIAS-3 and SOCS-3 expression and sensitizes human lung cancer xenograft in nude mice to paclitaxel. Oncotarget. (2015) 6:6386–405. doi: 10.18632/oncotarget.v6i8 PMC4467444 25788267

[91] Heppler LN Frank DA. Targeting oncogenic transcription factors: therapeutic implications of endogenous STAT inhibitors. Trends Cancer. (2017) 3:816–27. doi: 10.1016/j.trecan.2017.10.004 PMC5727919 29198438

[92] Wei J Costa C Ding Y Zou Z Yu L Sanchez JJ. mRNA expression of BRCA1, PIAS1, and PIAS4 and survival after second-line docetaxel in advanced gastric cancer. J Natl Cancer Institute. (2011) 103:1552–6. doi: 10.1093/jnci/djr326 21862729

[93] Chen P Zhao D Sun Y Huang L Zhang S Yuan Y. Protein inhibitor of activated STAT-1 is downregulated in gastric cancer tissue and involved in cell metastasis. Oncol Rep. (2012) 28:2149–55. doi: 10.3892/or.2012.2030 22972521

[94] Wang J Ni J Yi S Song D Ding M. Protein inhibitor of activated STAT xα depresses cyclin D and cyclin D kinase, and contributes to the inhibition of osteosarcoma cell progression. Mol Med Rep. (2016) 13:1645–52. doi: 10.3892/mmr.2015.4705 26708148

[95] Li C Boutet A Pascariu CM Nelson T Courcelles M Wu Z. SUMO proteomics analyses identify protein inhibitor of activated STAT-mediated regulatory networks involved in cell cycle and cell proliferation. Journal of proteome research (2023) 22:812–25. doi: 10.1021/acs.jproteome.2c00557 PMC9990128 36723483

[96] Mezencev R Mojzis J Pilatova M Kutschy P. Antiproliferative and cancer chemopreventive activity of phytoalexins: focus on indole phytoalexins from crucifers. Neoplasma. (2003) 50:239–45. 12937834

[97] Pedras MSC Montaut S Suchy M. Phytoalexins from the crucifer rutabaga: structures, syntheses, biosyntheses, and antifungal activity. J Organic Chem. (2004) 69:4471–6. doi: 10.1021/jo049648a 15202903

[98] Pilátová M Šarišský M Kutschy P Miroššay A Mezencev R Čurillová Z. Cruciferous phytoalexins: antiproliferative effects in T-Jurkat leukemic cells. Leukemia Res. (2005) 29:415–21. doi: 10.1016/j.leukres.2004.09.003 15725476

[99] Banerjee T Duhadaway J Gaspari P Sutanto-Ward E Munn DH Mellor AL. A key in *vivo* antitumor mechanism of action of natural product-based brassinins is inhibition of indoleamine 2, 3-dioxygenase. Oncogene. (2008) 27:2851–7. doi: 10.1038/sj.onc.1210939 18026137

